# Pathologic myopia and severe pathologic myopia: correlation with axial length

**DOI:** 10.1007/s00417-021-05372-0

**Published:** 2021-08-18

**Authors:** Ignacio Flores-Moreno, Mariluz Puertas, Elena Almazán-Alonso, Jorge Ruiz-Medrano, María García-Zamora, Rocío Vega-González, José M. Ruiz-Moreno

**Affiliations:** 1 Puerta de Hierro University Hospital, C/Manuel de Falla 1, 28222 Majadahonda (Madrid), Spain; 2 Department of Ophthalmology, Castilla La Mancha University, Albacete, Spain; 3 Miranza Corporation, Madrid, Spain; 4 Red Temática de Investigación Cooperativa en Salud: “Prevención, detección precoz, y Tratamiento de La Patología Ocular Prevalente, Degenerativa Y Crónica” (RD16/0008/0021), Spanish Ministry of Health, Instituto de Salud Carlos III, Madrid, Spain

**Keywords:** ATN classification, High myopia, Pathologic myopia, Severe pathologic myopia

## Abstract

**Purpose:**

This study had three aims: (1) correlate axial length (AL), age and best-corrected visual acuity in high myopic patients scored on the ATN grading system; (2) determine AL cut-off values to distinguish between pathologic myopia (PM) and severe PM; and (3) identify clinical differences between PM and severe PM.

**Methods:**

This is a cross-sectional, non-interventional study. All patients underwent complete ophthalmologic examination, ATN grading and multimodal imaging (colour fundus photography, swept-source OCT, fundus autofluorescence, OCT angiography and fluorescein angiography).

**Results:**

Six hundred forty-four eyes from 345 high myopic patients were included. The eyes were graded on the ATN system and classified as PM (≥ A2) or severe PM (≥ A3, ≥ T3 and/or N2). Significant between-group (PM vs. severe PM) differences (*p* < 0.05) were observed on the individual ATN components (atrophic [A], tractional [T] and neovascular [N]), age, BCVA and AL. AL was also linearly correlated with the A, T and N components (*r* = 0.53, *p* < 0.01; *r* = 0.24, *p* < 0.01; *r* = 0.20, *p* < 0.01; respectively). ROC curve analysis showed the optimal AL cut-off value to distinguish between PM at 28 mm (AUC ROC curve: 0.813, specificity: 75%, sensitivity: 75%) and severe PM at 29.50 mm (AUC ROC curve: 0.760, specificity: 75%, sensitivity: 70%).

**Conclusion:**

AL is the main variable associated with myopic maculopathy. Due to the clinical differences found between PM and severe PM, there is need to create an objective cut-off point to distinguish these two different entities being the optimal cut-off points for AL 28 mm and 29.5 mm, respectively. These objective AL cut-off values should be taken into account for determining a correct follow-up, ophthalmic management and treatment.

## Introduction


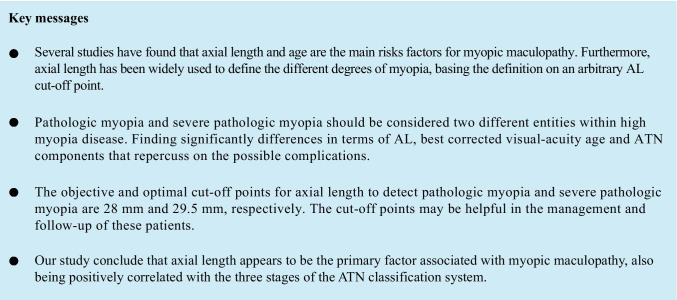


High myopia is a leading cause of central and peripheral vision loss, especially in Mediterranean and Asian countries [1, 2]. This condition is associated with various eye diseases, including glaucoma, retinal detachment, macular hole (MH), epiretinal membrane, choroidal neovascularization and chorioretinal atrophy. Most of these alterations are probably due to excessive elongation of the ocular globe, commonly associated with posterior staphyloma. Best-corrected visual acuity (BCVA) is worse in eyes with wide macular staphyloma compared to eyes without posterior staphyloma or other subtypes of staphyloma [3].

Ohno-Matsui et al. defined five categories of myopic maculopathy based on fundus colour photography (META-PM classification system), as follows: category 0, “no myopic retinal degenerative lesion”; category 1, “tessellated fundus”; category 2, “diffuse chorioretinal atrophy”; category 3, “patchy chorioretinal atrophy”; and category 4, “macular atrophy”. Three “plus” lesions were established: lacquer cracks, myopic choroidal neovascularization (CNV) and Fuchs spot [4]. Recently, a new classification system, known as the ATN grading system, was developed to assess the atrophic (A), tractional (T) and neovascular (N) components of myopic maculopathy [5]. The ATN classification system, which is based on fundus photography and optical coherence tomography (OCT) scans, has proven to be highly reproducible [6].

High myopia is usually defined as a spherical equivalent (SE) ≥  − 6 diopters (D) and/or axial length (AL) ≥ 26 mm. The definition of the term pathologic myopia (PM) was controversial until a group of experts defined it as highly myopic eyes with macular atrophic lesion equal to or more severe than diffuse chorioretinal atrophy, or the presence of posterior staphyloma [3, 4, 7]. Recently, the severe PM was defined according to ATN grading system [8], as follows: the presence of macular atrophic involvement ≥ A3 (patchy chorioretinal atrophy or macular atrophy), ≥ T3 (foveal detachment, full-thickness MH or MH plus retinal detachment) and/or N2 (active myopic CNV, scar CNV or Fuchs spot).

Macular pathology in high myopia can be very diverse in its presentation. Classifying it in high myopia, PM and severe PM will allow a better study, follow-up and treatment. Objective cut-off points will help in defining these conditions.

The aim of the present study was to evaluate a large sample of highly myopic eyes to assess the correlation between ATN scores and AL, posterior staphyloma, age, sex and BCVA. Actually, there is need to establish AL cut-off values for PM and severe PM, due to the fact that there are no quantitative objective criteria for defining these myopic statuses, and additionally, to identify differences in clinical characteristics between patients with PM and severe PM.

## Methods

This was a cross-sectional and non-interventional study in adhesion to the tenets of the Declaration of Helsinki for research involving humans. The study protocol was approved by the Ethics Committee of Puerta de Hierro-Majadahonda University Hospital and all included patients signed the appropriate informed consent. Highly myopic patients who visited Puerta de Hierro-Majadahonda University Hospital (Spain) from February 2017 to November 2020 were included.

Inclusion criteria were as follows: presence of high myopia (defined as AL ≥ 26 mm and SE ≥  − 6 D); clear ocular media; age ≥ 18 years; availability of good quality ocular images (> 45 image quality score on the DRI Triton Swept-Source OCT software [TOPCON Co., Japan]). Exclusion criteria were conditions of non-axial myopic eyes, previous intraocular surgery (exception for refractive surgery or cataract surgery); multifocal choroiditis; angioid streaks; punctate inner choroidopathy; coexisting ocular or systemic diseases including glaucoma; uveitis; diabetic retinopathy; retinal vein/artery occlusion; Marfan syndrome; or idiopathic MH. All the criteria were evaluated separately by two retinal experts and, in case of doubt, the eyes were excluded from the analysis.

Demographic data were obtained from the patients’ clinical records. All study participants underwent complete ophthalmological examination that included BCVA assessment under cycloplegia, slit-lamp anterior segment examination, optical biometry (IOL Master, Carl Zeiss, Tubingen, Germany), intraocular pressure (Goldmann applanation tonometry) and indirect fundus ophthalmoscopy.

Multimodal imaging included colour fundus photography, swept-source OCT (SS-OCT) and fundus autofluorescence (FAF) using DRI-OCT Triton plus. The structural OCT protocol included radial 12-mm scans centred at the fovea with 1,024 axial scans. Fluorescein angiography (FA) and OCT angiography (OCTA) were performed in cases of suspected myopic CNV. All examinations were performed in both eyes if the left and right eye both met the study inclusion criteria.

The ATN grading system [5, 6] for myopic maculopathy was applied in all cases. Two masked retinal specialists independently assessed the A, T and N components in each eye. The ATN system grades the A,T and N components, respectively, as follows: A0 (no myopic retinal lesions), A1 (tessellated fundus), A2 (diffuse chorioretinal atrophy), A3 (patchy chorioretinal atrophy) and A4 (complete macular atrophy); T0 (no macular schisis), T1 (inner or outer foveoschisis), T2 (inner and outer foveoschisis), T3 (foveal detachment), T4 (full-thickness MH) and T5 (MH + retinal detachment); and N0 (no myopic CNV), N1 (macular lacquer crack), N2a (active myopic CNV) or N2s (scar/Fuchs spot). N2a and N2s are dynamic stages, and both were considered N2 for statistical analysis (an eye classified as N2a before treatment can become N2s after treatment; N2s can start activity after time). In patients who had previously undergone pars plana vitrectomy for tractional disease, the presurgical T score was used (i.e., the impact of surgery on the traditional component was not considered).

Eyes with myopic CNV or myopic full-thickness MH were included in the study only if diffuse atrophy maculopathy was present in the atrophic maculopathy component to avoid including eyes with CNV or full-thickness MH secondary to other pathologies.

The presence of posterior staphyloma was based on fundus examination and OCT B-scan radial images. Two retinal experiences examiners determined the presence or absence of posteriori staphyloma.

All eyes were classified into two groups—PM or severe PM—based on ATN grading criteria described in previous studies [4, 8]. Briefly, PM was defined as the presence of an atrophic component ≥ A2 and severe PM was defined as myopic maculopathy ≥ A3, ≥ T3 and/or N2 (active myopic CNV, scar CNV or Fuchs spot).

### Statistical analysis

All analyses were performed using the IBM-SPSS statistical software program (IBM-SPSS, v. 26.0, Chicago, IL, USA). A two-tailed *p* value < 0.05 was considered statistically significant. Descriptive statistics were provided for normally distributed variables as means with standard deviation (SD) for quantitative variable and *n* (percentage) for categorical variables. The Kolmogorov–Smirnov test was performed to assess the normality or non-normality of the variables. Demographic data, BCVA, AL, SE, posterior staphyloma and ATN classification were compared between groups using independent *T* Student test for normally distributed variables and Chi-square test for normally distributed categorical variables. Pearson and Spearman correlations were used to determine the correlations for normally distributed and non-normally distributed variables, respectively. The results were expressed in terms of *r* and *p* value.

As Pearson’s and Spearman’s “*r* value” is interpreted, values between *r* = 0.30 and *r* =  − 0.30 are not considered clinically significant, being a weak or even null correlation (if *r* = 0).

Receiver operating characteristic (ROC) curves were prepared to obtain cut-off values using Youden’s index. Generalized estimating equation (GEE) multivariant analysis was performed to assess the effect of the A, T and N components with respect to AL, adjusting by age and controlling the other variables for each analysis.

## Results

A total of 644 eyes from 345 consecutive patients (69.3% women) with high myopia were included. The demographic characteristics of the full cohort are shown in Table 1.

**Table 1:** Demographic characteristics of the patient sample

Variable	Mean	Range	Standard deviation
Age, years	61.1	18–90	14.37
Axial length (mm)	29.5	26–37.6	2.48
BCVA (decimal; Snellen)	0.5; 20/40	0–1; 0–20/20	0.34; 20/60

*BCVA* best-corrected visual acuity

Eyes were classified into three groups according to their characteristics: (1) non-pathologic high myopic eyes, (2) PM group and (3) severe PM (subgroup of PM). Patients classified with severe PM were older and had longer AL and worse BCVA (*p* < 0.05) compared to the PM group. In addition, when comparing the latter with the total group of high myopes, differences were found in the three variables mentioned above (*p* < 0.01). The demographic and clinical characteristics are summarized in Table 2 according to the group classification (PM versus severe PM).

**Table 2:** Demographic characteristics of patients with pathologic myopia versus severe pathologic myopia

Variable	Pathologic myopia (*n* = 543)	Severe pathologic myopia (*n* = 278)	*P* value
Female	71.6% (389/543)	73.7% (205/278)	*p* > 0.05 (Chi-square test)
Male	28.3% (154/543)	26.2% (73/278)	*p* > 0.05 (Chi-square test)
Age ± SD (years)	62.98 ± 13.24	66.32 ± 12.14	*p* < 0.05 (*T* Student test)
Axial length ± SD (mm)	29.82 ± 2.44	30.78 ± 2.44	*p* < 0.05 (*T* Student test)
BCVA ± SD (decimal/Snellen)	0.49 ± 0.35 20/40 ± 20/60	0.35 ± 0.34 20/60 ± 20/60	*p* < 0.05 (*T* Student test)
SE ± SD (diopters)	− 12.52 ± 5.69	− 15.42 ± 5.66	*p* < 0.05 (*T* Student test)

*BCVA* best-corrected visual acuity; *SE* spherical equivalent; *SD* standard deviation

In terms of A, T and N components; there were statistically significant differences when comparing PM and severe PM group (*p* < 0.05). The number and percentage of each stage of A, T and N components is outlined in Table 3. On the other hand, when comparing the total PM group with the group of high myopia, statistically differences in ATN components were also found (*p* < 0.01).

**Table 3:** ATN scores for eyes with pathologic myopia versus severe pathologic myopia

A component			T component			N component		
	**PM**	**SPM**		**PM**	**SPM**		**PM**	**SPM**
**A0**	0% (0/543)	0% (0/278)	**T0**	45.6% (248/543)	43.5% (121/278)	**N0**	66.3% (360/543)	38.4% (107/278)
**A1**	0% (0/543)	0% (0/278)	**T1**	30.0% (163/543)	29.5% (82/278)	**N1**	3.5% (19/543)	2.8% (8/278)
**A2**	58.9% (320/543)	19.7% (55/278)	**T2**	19.7% (107/543)	17.9% (50/278)	**N2**	30.2% (164/543)	58.6% (163/278)
**A3**	27.6% (150/543)	53.9% (150/278)	**T3**	1.3% (7/543)	2.8% (7/278)			
**A4**	13.4% (73/543)	26.2% (73/278)	**T4**	2.9% (16/543)	5.7% (16/278)			
			**T5**	0.3% (2/543)	0.7% (2/278)			

*PM* pathologic myopia; *SPM* severe pathologic myopia

AL was linearly correlated with A and T components, as follows: A (*r* = 0.53, *p* < 0.01) and T (*r* = 0.34, *p* < 0.01). But the correlation with *N* score was clinically weak (*r* = 0.20, *p* < 0.01).

In addition to the correlation found regarding AL, there was also a moderate linear correlation between age and A score (*r* = 0.31, *p* < 0.01). However, not statistically or clinically relevant differences have been found regarding T and N scores (*r* = 0.09, *p* > 0.05; *r* = 0.17, *p* < 0.01, respectively). On the other hand, age was also negatively correlated with BCVA (*r* =  − 0.31, *p* < 0.01), but not statistically correlation was found between age and AL (*r* = 0.09, *p* > 0.05). BCVA was negatively correlated with the A (*r* =  − 0.45, *p* < 0.01) and N scores (*r* =  − 0.34, *p* < 0.01), but there was not proven a significant clinical correlation with the T component (*r* =  − 0.15, *p* < 0.01).

Posterior staphyloma was present in 47.7% eyes out of the total. Larger AL, older patients and also lower visual acuity (*p* < 0.01) were found in eyes with posterior staphyloma. Moreover, the presence of this hallmark of myopic retinal pathology was correlated with greater A and T components scores (*p* < 0.01). Nevertheless, no correlation was found when studying the correlation with N component (*p* > 0.05). In addition, the presence of posterior staphyloma in PM and severe PM groups was statistically significant (*p* < 0.01).

**Fig. 1:**
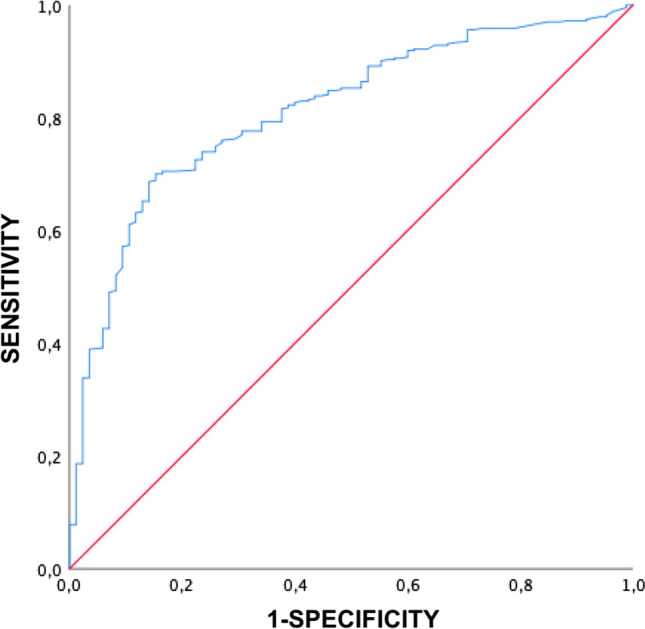
ROC curve of axial length in pathologic myopia group

**Fig. 2:**
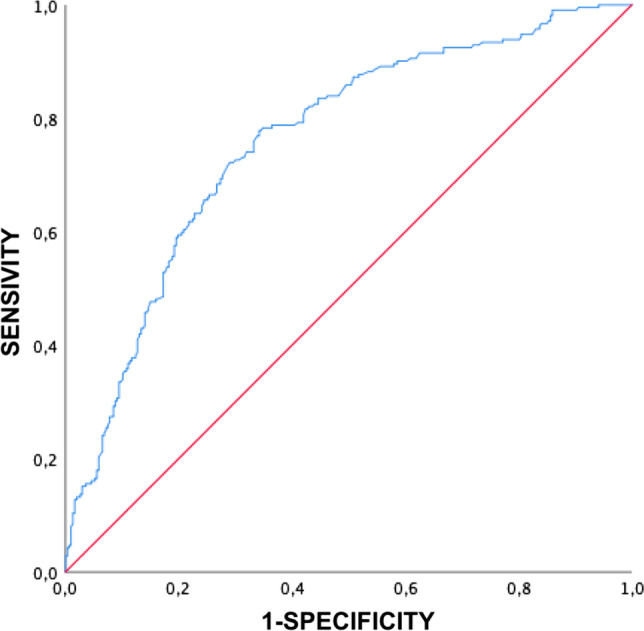
ROC curve of axial length in severe pathologic myopia group

On the ROC curve, the best AL cut-off point for the PM group, compared with non-pathologic high myopic eyes, was 28 mm (AUC: 0.813, 95% confidence interval: 0.767–0.856, specificity: 75%, sensitivity: 75%). For severe PM, compared with PM group and non-pathologic high myopic eyes, the cut-off point was at 29.50 mm (AUC: 0.760, 95% confidence interval: 0.718–0.802, specificity: 75%, sensitivity: 70%) (Figs. 1 and 2).

The age-adjusted association between ATN grade and AL was estimated on the multivariate GEE model, which showed no association between age and AL. Thus, age was not a confounding variable. The following differences in mean axial length were observed according to A grade: grade A2 eyes were 2.17 mm longer than A0 (*p* < 0.01), A3 were 3.36 mm longer than A0 (*p* < 0.01), and A4 were 3.47 mm longer than A0 (*p* < 0.01). All of these analyses and results were adjusted for the T and N variables.

By T grade, the following differences in AL were observed: grade T1 eyes were 0.33 mm longer than T0 (*p* < 0.05), T1 were 0.74 mm longer than T0 (*p* < 0.05), and T1 were 1.16 mm longer than T0 (*p* < 0.05). The T3 and T5 groups did not show any statistically significant differences (both *p* > 0.05). No statistically significant differences in AL were observed between any of the N groups (*p* > 0.05; N2 *p* > 0.05).

The Cohen’s kappa coefficients between graders by component (A, T, N) were as follows: A: 0.97 (*p* < 0.01); T: 0.97 (*p* < 0.01); and N: 0.99 (*p* < 0.01).

## Discussion

This study shows the importance of establishing objective cut-off points for these entities within high myopia, due to the differences shown by the objective parameters analysed in these groups and the consequent clinical repercussion in these patients and in their clinical management.

In this study, axial length was positively correlated with the A, T and N components of the ATN grading system. BCVA was negatively correlated with all three ATN components. Age was positively correlated with the A stage, but no correlation was found neither with T nor N component. On the ROC curve analysis, the optimal AL cut-off values to detect PM and severe PM were 28 mm and 29.5 mm, respectively.

Several studies have found that age is a key risk factor for macular complications in patients with highly myopic eyes or PM [9–12]. Progression rates have been well-documented in studies with long follow-up. Fang et al. [10] used colour fundus retinography to assess patients with myopic maculopathy (mean follow-up: 18 years), showing that 58.6% of eyes progressed, at a rate of 47.0 per 1000 eye-years. The progression rate was higher in eyes with the most advanced A component stage of myopic maculopathy (macular atrophy and patchy atrophy). Yokoi et al. [13] evaluated the eyes of myopic children and followed them into adulthood (minimum follow-up: 20 years), concluding that adult patients who presented features of PM had previously presented pathologic myopic changes during childhood. In addition, the presence of peripapillary diffuse atrophy in childhood was considered a biomarker for future myopic chorioretinal atrophy. Those authors also observed progression in the stages of atrophic myopic maculopathy and an extension of the atrophic areas across the entire macula over the course of follow-up.

In a population-based study involving patients > 48 years of age, Vongphanit et al. [14] observed a non-significant trend towards an association between myopic maculopathy and age. In our series, consistent with previous reports, age was positively correlated with the A stage (ATN classification), indicating that older myopic patients with some risk factors were more likely to present more advanced stages of atrophic maculopathy. Over time, diffuse and patchy atrophy areas are expected to increase in size, potentially coalescing into larger chorioretinal atrophic patches.

Age was not correlated with either the T or N components of the ATN system. Given that myopic tractional maculopathy (MTM) is due to the relationship between the posterior hyaloid and posterior shape of the globe, it is reasonable to expect that the association between age and posterior staphyloma would have an important impact on the development of tractional maculopathy. However, given that neither our study nor Matsumura et al. [15] found any correlation between age and T stage, it appears that axial length may have a stronger effect than age on the development of tractional maculopathy.

Myopic CNV is the main and most common aetiology for CNV in patients ≤ age 50 [16, 17]. Chen et al. [18], in a large sample of eyes (*n* = 820) graded according to the ATN system, found no association between age and N score (ATN grading system), a finding that is consistent with our results. Wakabayashi et al. [19] evaluated the possible risk factors for myopic CNV. In that study, the univariate analysis showed a correlation between age and CNV; however, based on the results of the logistic regression analysis, the authors hypothesized that ageing may be responsible for the choroidal filling delay in FA and for choroidal thinning, which are the main factors associated with myopic CNV.

In our study, AL was the main factor associated with myopic maculopathy in the A, T and N stages. These results are consistent with previous studies that assessed the correlations between AL and the atrophic components of myopic maculopathy [9–12, 14, 18, 20]. Xiao et al. found that the odds of developing myopic atrophic maculopathy (multivariate model) was 2.97 times higher for each millimetre increase in AL [9]. From our results, significant between-group differences in AL according to the A grade (ATN system) were observed, as follows: A2 (diffuse atrophy), 2.17 mm; A3 (patchy atrophy), 3.36 mm; and A4 (complete macular atrophy), 3.47 mm. Those findings reveal significant differences in AL among eyes considered PM [4] (≥ A2), thus indicating the clinical importance of AL in detecting PM.

Studies have shown that MTM is more common in eyes with AL > 28 mm [15]. Two studies conducted in Asia found a positive association between MTM, AL and higher degrees of myopia [15, 21]. Shimada et al. [22] studied the natural course and risk factors for MTM in a sample of patients with ≥ 24 months of follow-up. There were no differences in AL across the baseline tractional groups and no differences in AL between the groups that improved, were stable or progressed. However, that study consisted of patients with early stage MTM. Classification was made according to the extension of retinoschisis in the macular area. A statistically significant difference was observed in the tractional stage compared to T0 since T1, with a variation of 0.33 mm, in T2 of 0.74 mm. No differences were found for T3 (foveal detachment), but T4 (full-thickness MH) showed a statistical difference of 1.16 mm. One hypothesis put forth to explain this finding is that the two stages do not differ in terms of AL, which suggests that other factors are likely responsible for generating foveal detachment or directly causing full-thickness MH. While no significant differences in AL in stage T5 were observed, only two patients were classified as T5, although it is worth noting that one of these T5 patients had the longest AL in the entire sample.

Taking into account AL and the neovascular stage of myopic maculopathy, it has to be remarked that most of the studies employed just colour fundus photography to study myopic maculopathy. In our study, SS-OCT was used, which has a higher sensitivity to detect and monitor myopic CNV (up to 97%) [23]. In the study by Chen et al., atrophic and neovascular maculopathy were both associated with the same risk factors, which were mainly longer AL, larger peripapillary atrophy and thinner macular choroidal thickness [18]. Other studies have found that AL is a prognostic factor for final BCVA in young patients with myopic CNV [24].

The development of posterior staphyloma is a key factor for myopic macular maculopathy. Almost half of the eyes included in our study had posterior staphyloma and there was a correlation with longer AL, worse BCVA and greater A and T component of the ATN classification system and posterior staphyloma was statistically more frequent in PM and severe PM compared to high myopia.

PM and severe PM have been defined to asses myopic conditions associated with decreased vision [7, 8]. In the present study, the group of patients classified with severe PM consisted mainly of older patients with longer AL and worse BCVA. Moreover, these groups (PM versus severe PM) differed significantly on all three ATN components. AL has been widely used to define the degree of myopia, with some studies defining high myopia based on an arbitrary AL cut-off point of 26 or 26.5 mm [5, 25, 26]. The ROC curve analysis in our study suggests that the optimal AL cut-off points to define PM and severe PM are 28 and 29.5 mm, respectively, with good sensitivity and specificity. Based on these findings, the following criteria could be used to define high myopia: AL ≥ 26 mm, PM ≥ 28 mm and severe PM ≥ 29.5 mm. These cut-off points would help to establish myopic conditions numerically by relying on the clinical features defined previously [4, 8].

This study has several limitations. First, ATN grading was based on a subjective assessment of colour fundus photography and SS-OCT images by two investigators; nevertheless, based on the excellent Cohen kappa coefficients, it suggests that the grading was reliable. Second, due to the sample characteristics, relatively few eyes were classified as T3 and T5, which could affect the strength of the statistical analysis, as could be the case regarding correlation between BCVA and T component. Moreover, extrapolation must be done carefully, taking into account the sample size; studies with large sample sized of these subgroups should be carried out. Third, posterior staphyloma was not evaluated using wide-field colour fundus, wide-field OCT or ocular MRI. Future studies including these technologies would be necessary. Fourth, due to the cross-sectional study design, AL and myopic maculopathy progression over time were not able to assess; consequently, a prospective study is needed to confirm our findings.

In conclusion, axial length appears to be the primary factor associated with myopic maculopathy being correlated with the three components of the ATN classification system. Interestingly, age was positively correlated with A component, but it was not correlated with either the T or N components; this supports the stronger influence of axial length on the development of myopic tractional maculopathy. There is need to create an objective cut-off point to distinguish between PM and severe PM. The optimal cut-off points for axial length to detect PM and severe PM are 28 mm and 29.5 mm, respectively. Eyes classified as PM or severe PM differed significantly in terms of patient AL, BCVA, age and ATN components with direct repercussions on the possible complications and management of these patients. For this reason, two different entities should be taken into account objectified by AL cut-off values for closer follow-up, ophthalmic management and treatment.

## Data Availability

The author declares that data is available upon justified request.
